# The utility of free-breathing, motion-corrected late gadolinium enhancement for right ventricular fibrosis imaging in congenital heart disease

**DOI:** 10.1186/1532-429X-17-S1-P221

**Published:** 2015-02-03

**Authors:** Ee Ling Heng, Peter Kellman, Raad Mohiaddin, Francisco Alpendurada, Philip J Kilner, Dudley J Pennell, Michael A Gatzoulis, Jennifer Keegan, Sonya V Babu-Narayan

**Affiliations:** NIHR Cardiovascular Biomedical Research Unit, Royal Brompton Hospital, London, UK; National Heart & Lung Institute, Imperial College London, London, UK; National Heart, Lung, and Blood Institute, National Institutes of Health, Bethesda, MD USA; Cardiovascular Magnetic Resonance Unit, Royal Brompton Hospital, London, UK; Department of Adult Congenital Heart Disease, Royal Brompton Hospital, London, UK

## Background

Ventricular fibrosis in adult congenital heart disease (ACHD) has been shown to relate to adverse clinical markers.[1, 2] Novel free-breathing, motion-corrected late gadolinium enhancement cardiovascular magnetic resonance (LGE CMR) has been shown to be advantageous in quantifying LV LGE, but has not been evaluated for RV LGE. [3] It may therefore also be beneficial in ACHD patients, particularly with RV LGE patterns. We sought to investigate its clinical utility in ACHD patients undergoing LGE CMR.

## Methods

Ten adult repaired Tetralogy of Fallot patients aged ≥16 years with glomerular filtration rate>30ml/min completed clinical CMR scans acquired by a single operator on a 1.5T Siemens Avanto for volumetric and functional biventricular assessment. LGE imaging was performed 10 minutes after 0.1mmol/kg intravenous gadobutrol administration (Bayer, Germany).

Interspersed breath-hold (BH) segmented gradient echo and free-breathing (FB) motion-corrected balanced steady state free precession phase-sensitive inversion recovery (PSIR) pulse sequences were acquired with identical typical imaging parameters of: field of view 380x320mm, matrix 256x144, 6mm slice thickness with 4mm gap for short-axis stacks, and acceleration factor (GRAPPA) 2. Repetition time/echo time of 8.3/3.8ms (BH) 2.7/1.7ms (FB), flip angle 25^o^ (BH) and 50^o^ (FB), pixel bandwidth 140Hz (BH) 977Hz (FB) with acquisition window 166ms (BH) 196ms (FB) were set. Inversion times were adjusted for myocardial nulling throughout, and kept constant between both sequences when acquiring identical slice locations in long and short-axis views. When inversion time drift was minimal, transverse navigator-gated 3D LGE imaging was performed as per published protocol as a reference standard.^4^

Anonymised, blinded image analysis was carried out by two experienced observers assessing RV LGE image quality,^5^ image confidence scores and semi-quantitative RV LGE scoring.^1^Blood-myocardium and Gd-myocardium contrast-to-noise ratios (CNRs) were also compared.

## Results

**Figure 1:**
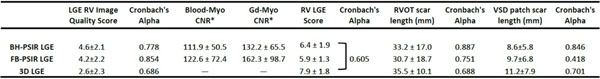
Blinded RV LGE quality image and semi-quantitative analysis. LGE RV Image Quality scores adapted from Euro-CMR registry standardised parameters (10 criteria, range of score 0-19 with lower scores denoting better image quality).^5^ RV LGE score reflects extent of fibrosis as previously published (range 0-20 with higher scores indicating greater fibrosis burden).^1^ Interobserver agreement was assessed with Cronbach's alpha. BH: Breath-hold FB: Free-breathing CNR: Contrast-to-noise ratio. *For the purposes of this abstract, CNR was estimated as the difference in signal intensity divided by the standard deviation of the signal intensity in a background ROI (that is, no consideration was made for parallel imaging).

**Figure 2:**
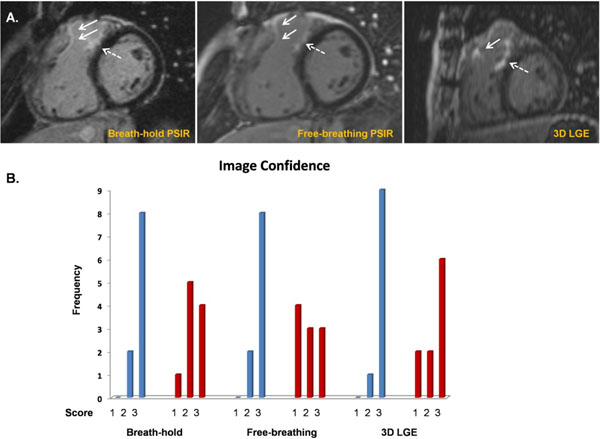
(A) Short-axis view of typical RV LGE pattern in repaired Tetralogy of Fallot patients with RVOT patch fibrosis (white arrows) and VSD patch fibrosis (dotted white arrows). (B) Observer image confidence scores (1 = low 2 = some 3 = high confidence) with blue bars representing Observer 1 and red bars representing Observer 2.

RV LGE was present in all patients and LV LGE in 7 patients (at LV apex from previous surgical apical vent). There was reasonable interobserver agreement when scoring the extent of RV LGE (α=0.605) with good overall agreement in quantifying the extent of RVOT and VSD patch scar (Figures 1 and 2). Image quality scores were comparable for BH and FB sequences (4.6±2.1 vs 4.2±2.2, p=0.55), with better image quality scores for 3D LGE when compared to 2D PSIR LGE (p<0.05). Blood-myocardial and Gd-myocardial CNRs were higher for FB PSIR in relation to BH PSIR.

## Conclusions

Free-breathing LGE provides comparable, diagnostic image quality to conventional 2D BH LGE imaging and may be practical for clinical use, especially for patients unable to breath-hold or with arrhythmias that would otherwise compromise image quality.

## Funding

British Heart Foundation, NIHR Cardiovascular Biomedical Research Unit of Royal Brompton & Harefield NHS Foundation Trust and Imperial College London.
